# Circulating microRNAs as novel potential diagnostic biomarkers for ovarian cancer: a systematic review and updated meta-analysis

**DOI:** 10.1186/s13048-019-0482-8

**Published:** 2019-03-21

**Authors:** Xinshuai Wang, Dejiu Kong, Chaokun Wang, Xuezhen Ding, Li Zhang, Mengqi Zhao, Jing Chen, Xiangyun Xu, Xiaochen Hu, Junqiang Yang, Shegan Gao

**Affiliations:** Henan Key Laboratory of Cancer Epigenetics, Cancer Hospital, The First Affiliated Hospital, College of Clinical Medicine, Medical College of Henan University of Science and Technology, Luoyang, 471003 China

**Keywords:** microRNAs, Ovarian cancer, Diagnosis, Multiple miRNA panels, Meta-analysis

## Abstract

**Object:**

Ovarian cancer is the primary cause of cancer-associated deaths among gynaecological malignancies. Increasing evidence suggests that microRNAs may be potential biomarkers for the diagnosis and prognosis of cancer. In this study, we conducted a systematic review and meta-analysis to summarize the global research and to evaluate the overall diagnostic accuracy of miRNAs in detecting ovarian cancer.

**Methods:**

A systematic literature search was conducted for relevant studies through July 20, 2017, in English databases (CENTRAL, MEDLINE, and EMBASE), the Grey reference database and Chinese databases. Statistical analysis was conducted using OpenMetaAnalyst, STATA 14.0 and RevMan 5.3. Pooled sensitivity, specificity, and other parameters were used to assess the overall miRNA assay performance using a bivariate random-effects model (BRM). Meta-regression and subgroup analyses were performed to dissect the heterogeneity. Sensitivity analysis was performed to assess the robustness of our analysis, and the publication bias of the selected studies was assessed using Deeks’ funnel plot asymmetry test.

**Results:**

Thirteen articles described 33 studies, including 1081 patients with ovarian cancer and 518 controls. The pooled results were as follows: sensitivity, 0.89 (95% CI: 0.84–0.93); specificity, 0.64 (95% CI: 0.56–0.72); positive likelihood ratio, 2.18 (95% CI: 1.89–2.51); negative likelihood ratio, 0.15 (95% CI: 0.11–0.22); and diagnostic odds ratio (DOR), 13.21 (95% CI: 9.00–19.38). We conducted subgroup analyses based on ethnicity, research design, and miRNA profiling and found that multiple miRNA panels were more accurate in detecting ovarian cancer, with a combined DOR of 30.06 (95% CI: 8.58–105.37).

**Conclusion:**

Per the meta-analysis, circulating miRNAs may be novel and non-invasive biomarkers for detecting ovarian cancer, particularly multiple miRNA panels, which have potential diagnostic value as screening tools in clinical practice.

**Electronic supplementary material:**

The online version of this article (10.1186/s13048-019-0482-8) contains supplementary material, which is available to authorized users.

## Background

Per the International Agency for Cancer Research, Cancer Statistics 2016, ovarian cancer is the fifth most common cause of cancer-related deaths in the United States, with an estimated 22,280 new cases and 14,240 deaths in 2016 [1]. In China, ovarian cancer incidence and mortality have increased over the past 10 years [2, 3], and ovarian cancer is prone to early metastasis during its progression. Most patients with ovarian cancer are not diagnosed until the cancer has advanced, and the 5-year survival rate for patients with advanced ovarian cancer is only 30% due to recurrence and drug resistance [4, 5]. In the early stages of ovarian cancer, tumours are confined to the ovary and are more likely to be cured, which sharply increases the 5-year survival rate to 92.7% [6]. Thus, appropriate biomarkers are urgently needed for early screening and diagnosing ovarian cancer in clinical practice to improve overall survival. Recent studies have demonstrated that microRNAs are important in many physiological processes, including regulating cell growth, apoptosis, metastasis, drug resistance, and ovarian cancer invasion [7–9].

MicroRNAs (miRNAs) are a class of small RNA molecules (19–22 nucleotides) that negatively regulate gene expression by inhibiting translation or degrading the target mRNA [10]. MiRNA is stably expressed in plasma, serum and other body fluids, indicating its potential as a clinical biomarker. As research progresses, increasing evidence suggests that miRNAs may be biomarkers for the diagnosis and prognosis of cancer [11–14]. Although different miRNA family members exhibit differential biological characteristics in cancer development, the same miRNA research may have conflicting results. Richer et al. suggested that miR-200c is both a “guard epithelial phenotype” and a gene that inhibits epithelial-to-mesenchymal direct targeting inhibition [15]. Cao et al. revealed that miR-200c overexpression may promote aggressive tumour progression and thus be a reliable marker for predicting EOC patient survival [16].

Currently, several groups have reported the potential diagnostic value of circulating miRNAs in various cancers such as oesophageal cancer, head and neck tumours, and gastric cancer [17–19]; however, microRNA’s diagnostic value for ovarian cancer remains unclear. The results are inconsistent due to differences in study design, specimen types, and miRNAs, and different groups have even obtained conflicting conclusions.

In this study, we conducted a systematic review and meta-analysis to summarize global research and to evaluate the overall diagnostic accuracy of miRNAs in detecting ovarian cancer. We also explored an optimal miRNA and a potential panel to improve its diagnostic performance.

## Material and methods

### Search strategy

The selected databases were searched through July 20, 2017, and included Chinese databases (e.g., China National Knowledge Infrastructure/Chinese Wanfang Database/Chinese VIP Database), English databases such as CENTRAL (through thecochranelibrary.com), MEDLINE (through pubmed.gov), and EMBASE (through ovidsp.tx.ovid.com) and the Grey Reference database (e.g., OpenGrey/WHO-ICTRP). We used the search strategy of subject headings and random words, which was designed by two researchers and included the following terms: (“miRNAs” OR “microRNAs” OR “miR*”) AND (“ovarian cancer” OR “ovary cancer” OR “ovarian neoplasms”) AND (“diagnos*” OR “ROC curve” OR “sensitivity” OR “specificity”). Search details are shown in Additional file 1.

### Study inclusion/exclusion criteria

**Fig. 1:**
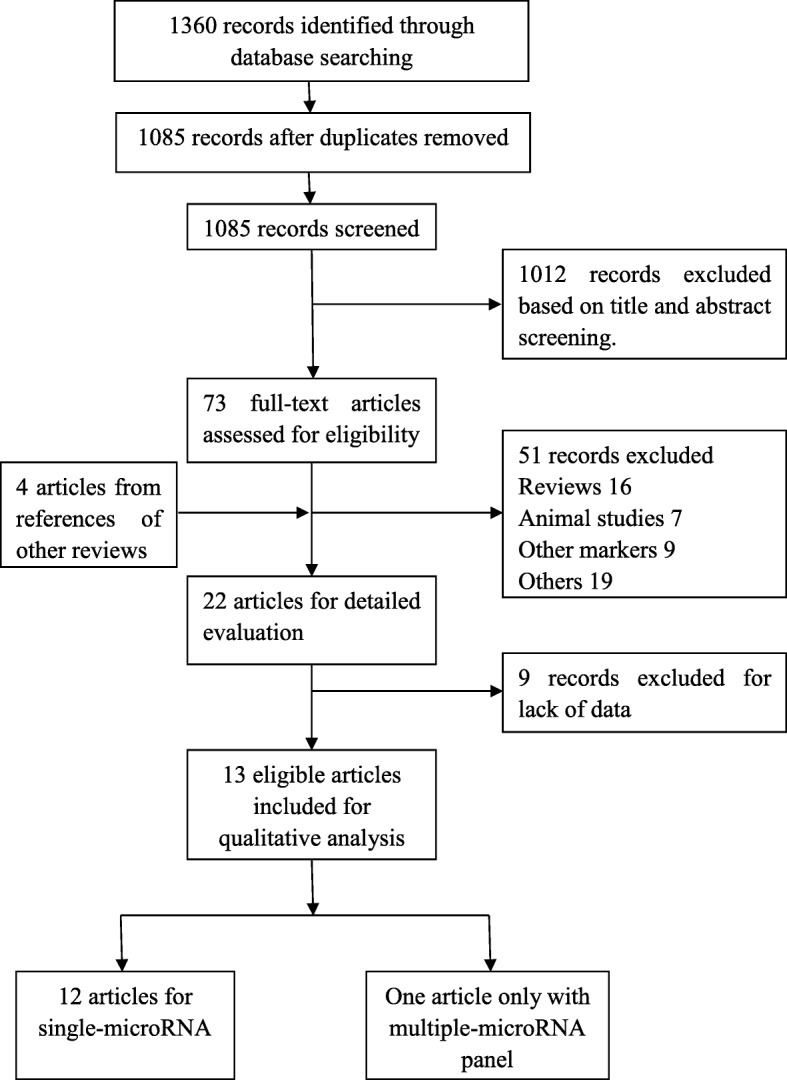
Flow Diagram in our study

Studies were considered eligible if the publications met all the following criteria: (1) studies were diagnostic and used circulating miRNAs; (2) histological subtype was specified as ovarian cancer; (3) sufficient data were reported to generate a 2 × 2 table for calculating true positives (TP), false positives (FP), false negatives (FN), and true negatives (TN); and (4) studies were not reviews, abstracts, animal studies or editorial articles. Studies were excluded for the reasons described in Fig. 1 (Flow Diagram).

### Data extraction

Eligible articles were reviewed independently by two reviewers (DJK and CKW), and disagreements were resolved by consensus. The necessary information and data were extracted from the final eligible articles as follows: first author, year of publication, country of origin, subjects’ ethnicity, number of cases and controls, research types, median age, miRNA expression test methods, specimens, cut-off values, expression changes, and data needed for diagnostic meta-analyses (sensitivity and specificity) (Table 1).

**Table 1:** Characteristics of the studies included in the meta-analysis

ID	First Author	Year	Country	Median age	Type (Retro/Pro)	Cancer	Control	Standard	Specimen	Method	TNM stage (I-II/III-IV)	microRNAs	TN	TP	FN	FP	Cut-off	Level
1	Todeschini	2017	Italy	61	Pro	168	65	Yes	serum	qPCR	0/168	miR-1246	50	146	15	22	0.41	high
												miR-595	55	79	10	89	−0.29	high
												miR-2278	43	136	22	32	0.26	high
												3 miRNAs	50	141	15	27	0.41	high
2	Zuberi(a)	2016	India	NA	Pro	70	70	Yes	serum	qPCR	33/37	miR-125b	54	44	16	26	3.76	high
3	Zuberi(b)	2016	India	NA	Pro	70	70	Yes	serum	qPCR	33/37	miR-199a	67	48	3	22	4.71	high
4	Meng(a)	2016	Germany	60	Pro	163	20	Yes	serum	qPCR	27/118	miR-200a	18	137	2	26	NA	high
												miR-200b	20	86	0	77	NA	high
												miR-200c	20	51	0	112	NA	high
												3 miRNAs	18	144	2	19	NA	
5	Meng(b)	2016	Germany	56	Pro	60	32	Yes	serum	qPCR	8/41	miR-200a	29	40	3	20	NA	high
												miR-200b	29	56	3	4	NA	high
												miR-200c	27	51	5	9	NA	high
												3 miRNAs	32	50	0	10	NA	
6	Meng(c)	2015	Germany	62	Pro	180	66	Yes	serum	qPCR	32/147	miR-7	49	96	17	84	NA	high
												miR-25	42	166	24	14	NA	low
												miR-93	41	158	25	22	NA	low
												miR-429	63	107	3	73	NA	high
						72	66				NA	4 miRNAs	61	67	5	5	NA	
7	Gao	2015	China	NA	Retro	93	50	Yes	serum	qPCR	54/20	miR-200c	35	67	15	26	NA	high
												miR-141	36	64	14	29	NA	high
8	Zheng	2013	China	53.75	Retro	134	70	Yes	plasma	qPCR	45/89	miR-205	66	40	4	94	NA	high
9	Suryawanshi	2013	USA	65.52	Retro	21	20	Yes	plasma	qRT-PCR	3/18	miR-16, 191, and 4284	11	19	9	2	NA	high
10	Hong	2013	China	58.6	Retro	96	35	Yes	serum	qRT-PCR	32/64	miR-221	5	85	30	11	NA	high
11	Guo	2013	China	54.5	Retro	50	50	Yes	serum	qRT-PCR	35/15	miR-92	38	40	12	10	NA	high
12	Chung	2013	Korea	57.5	Retro	18	12	Yes	serum	qPCR	3/14	miR-132	11	12	1	6	0.0001	low
												miR-26a	9	18	0	3	0.0041	low
												let-7b	12	15	3	0	0.066	low
												miR-145	12	9	9	0	0.0011	low
13	Kan	2012	Australia	63	Retro	28	28	Yes	serum	qPCR	1/27	miR-200a	10	24	18	4	NA	high
												miR-200b	10	24	18	4	NA	high
												miR-200c	16	20	12	8	NA	high
												miR-200b + 200c	13	22	15	6	NA	high

*Retro* retrospective study, *Pro* prospective study, *qPCR* quantitative real-time PCR, *qRT-PCR* quantitative reverse transcription–PCR, *TN* true negatives, *TP* true positives, *FN* false negatives, *FP* false positives; level refers to the high or low levels of miRNA expression in blood compared with normal tissues.

### Trial quality assessment

The methodological qualities of the selected eligible articles were assessed by the Quality Assessment of Diagnostic Accuracy Studies 2 (QUADAS-2) score system. The QUADAS-2 tool combines the patient selection index, index test, reference standard, and flow and timing to evaluate risk of bias and applicability. The 7 items (four items on bias risk and three items on applicability) were assessed for all included articles. Two authors independently tested the pilot QUADAS-2 items (DJK and LZ), and discrepancies were resolved by a third author (XSW).

### Statistical analysis

Data were analysed using OpenMetaAnalyst (open-source, cross-platform software for advanced meta-analysis), Stata statistical software (v.14.0; Stata Corp, USA) and RevMan 5.3 (Cochrane Collaboration, Oxford, UK). The pooled parameters sensitivity, specificity, positive likelihood ratio (PLR), negative likelihood ratio (NLR), and diagnostic odds ratio (DOR) and their 95% CIs were calculated to evaluate the overall diagnostic accuracy. A bivariate random effects model (BRM) was also used, and the correlation value was applied to evaluate threshold effects by BRM. In addition, a χ^2^-based Cochran’s Q test was performed to assess the heterogeneity of the results across studies. If *P* < 0.10 and I^2^ was over 50%, it was considered heterogeneous, and meta-regression and subgroup analyses were performed to dissect the heterogeneity by the included studies’ characteristics. Sensitivity analysis was performed to assess the robustness of our analysis, and the publication bias of the selected studies was assessed using Deeks’ funnel plot asymmetry test.

## Results

### Included studies

The flow diagram of the selected studies is depicted in Fig. 1. The initial literature search identified 1360 articles, from which 275 duplicates were excluded. Of the remaining 1085 articles, 1012 records were excluded based on title and abstract screening. The search identified 73 full texts, of which 51 articles were excluded for the following reasons: (1) studies were not ovarian cancer diagnostic research; (2) lack of sufficient sensitivity and specificity data; (3) the markers were not microRNAs; (4) studies were animal studies, conference abstracts, prognostic studies, reviews or meta-analyses; and (5) cancer sample size was < 10. Additionally, we found four potential articles from the references of other reviews in the full-text screening process (Fig. 1). Finally, 13 articles were included in the diagnostic meta-analysis per the inclusion and exclusion criteria (Table 1) [20–32].

### Study characteristics and quality assessment

**Fig. 2:**
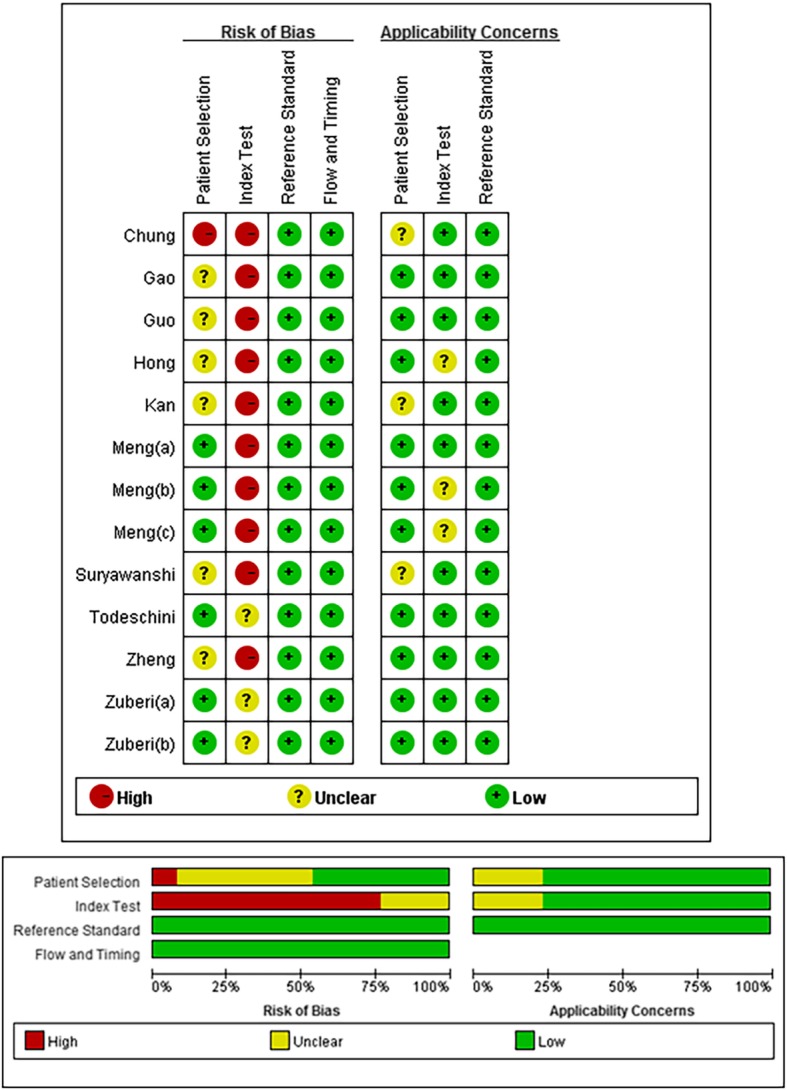
Bias risk and applicability concerns summary and graph

Table 1 shows the characteristics of eligible studies published between 2012 and 2017. Single-miRNA assays and multiple-miRNA panel assays were included, and the test results were considered as separately grouped data. If an article contained two independent tests (discovery group and validation group), the result was treated as two datasets [25]. The 13 articles comprised 33 studies, including 1081 ovarian cancer patients and 518 controls. In all included studies, ovarian cancer was diagnosed pathologically, which is considered the gold standard for diagnosing ovarian cancer. From the 13 diagnostic articles, several countries and regions were included: seven focused on Asia; four focused on Europe; and the other two articles focused on the United States and Australia. Five articles included both single-miRNA assays and multiple-miRNA panel assays [20, 23–25, 32]. One article used multiple-microRNA panel assays only [28], while the remaining 7 investigated the diagnostic performance of multiple miRNAs. The quality evaluation results based on QUADAS-2 using RevMan 5.3 are shown in Fig. 2 and indicate that all studies had moderately high scores, and relatively high quality studies were included. As a result, 33 datasets from 13 articles were analysed in the quantitative synthesis.

### Diagnostic accuracy of circulating miRNAs in ovarian cancer

**Fig. 3:**
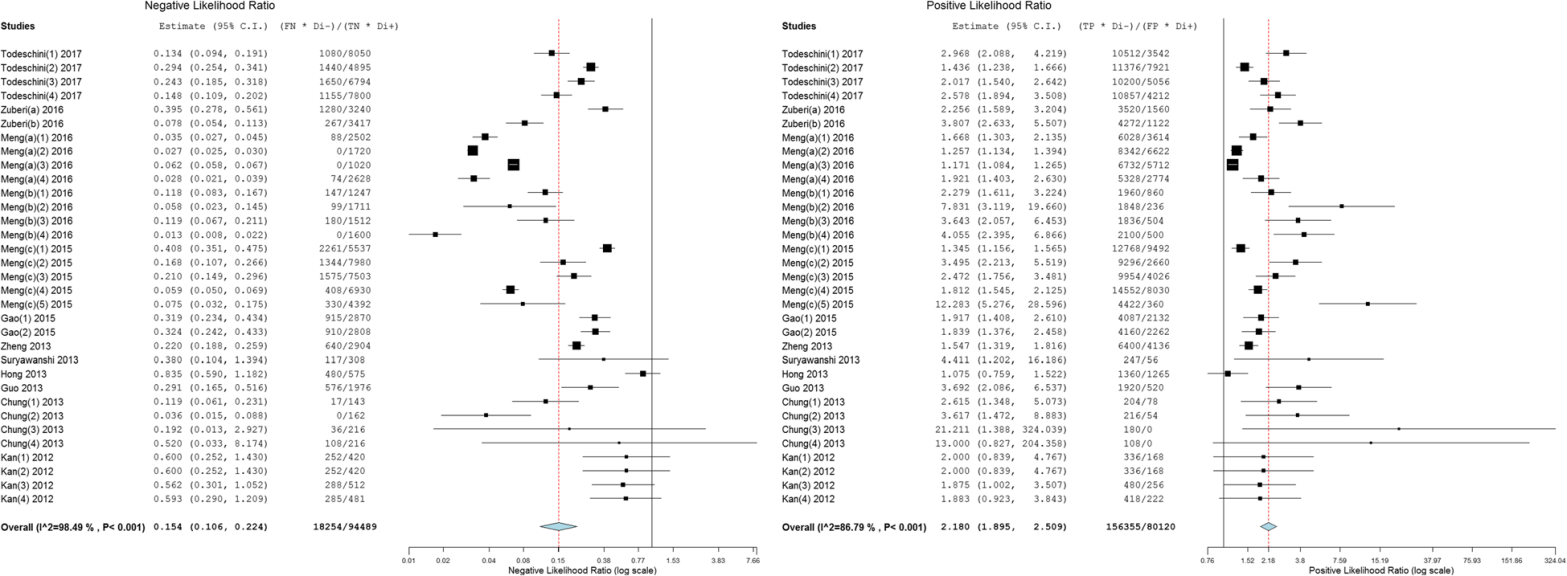
Forest plots of the negative likelihood ratio (NLR) and positive likelihood ratio (PLR) of the miRNA assays

**Fig. 4:**
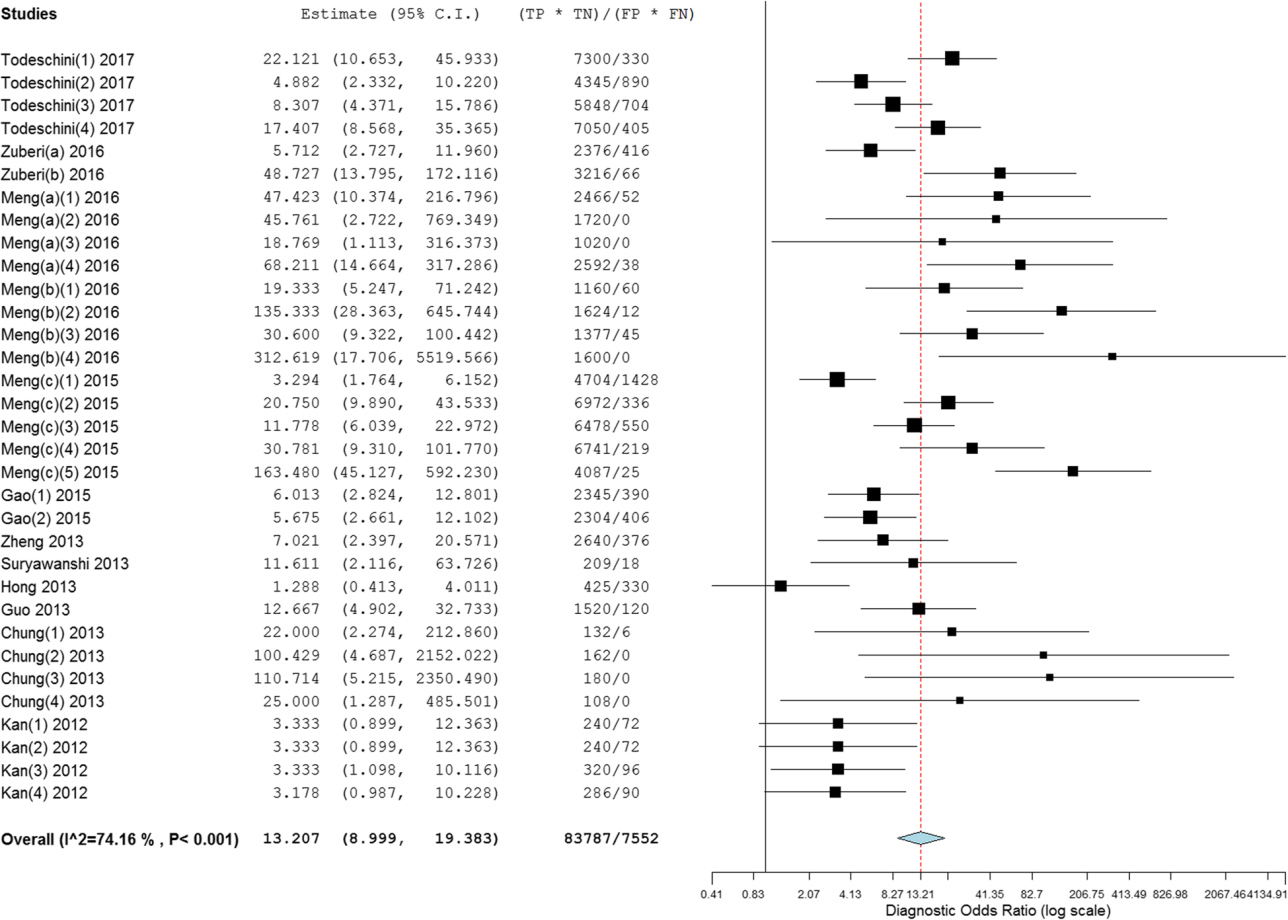
Forest plots of the diagnostic odds ratio (DOR) of the miRNA assays

As shown in Figs. 3 and 4, forest plots of NLR, PLR and DOR for the miRNA assays used to detect ovarian cancer were constructed by OpenMetaAnalyst with BRM, and the heterogeneity analysis revealed I^2^ values of 98.49% (*P* < 0.001) for NLR, 86.79% (*P* < 0.001) for PLR and 74.16% (*P* < 0.001) for DOR, indicating significant heterogeneity; thus, the random-effects model was selected. The overall pooled results for sensitivity and specificity were 0.89 (95% CI: 0.84–0.93) and 0.64 (95% CI: 0.56–0.72), indicating moderate accuracy. The overall DOR, PLR, and NLR were 13.21 (95% CI: 9.00–19.38), 2.18 (95% CI: 1.89–2.51) and 0.15 (95% CI: 0.11–0.22), also indicating moderate accuracy.

### Heterogeneity test and subgroup analysis

**Fig. 5:**
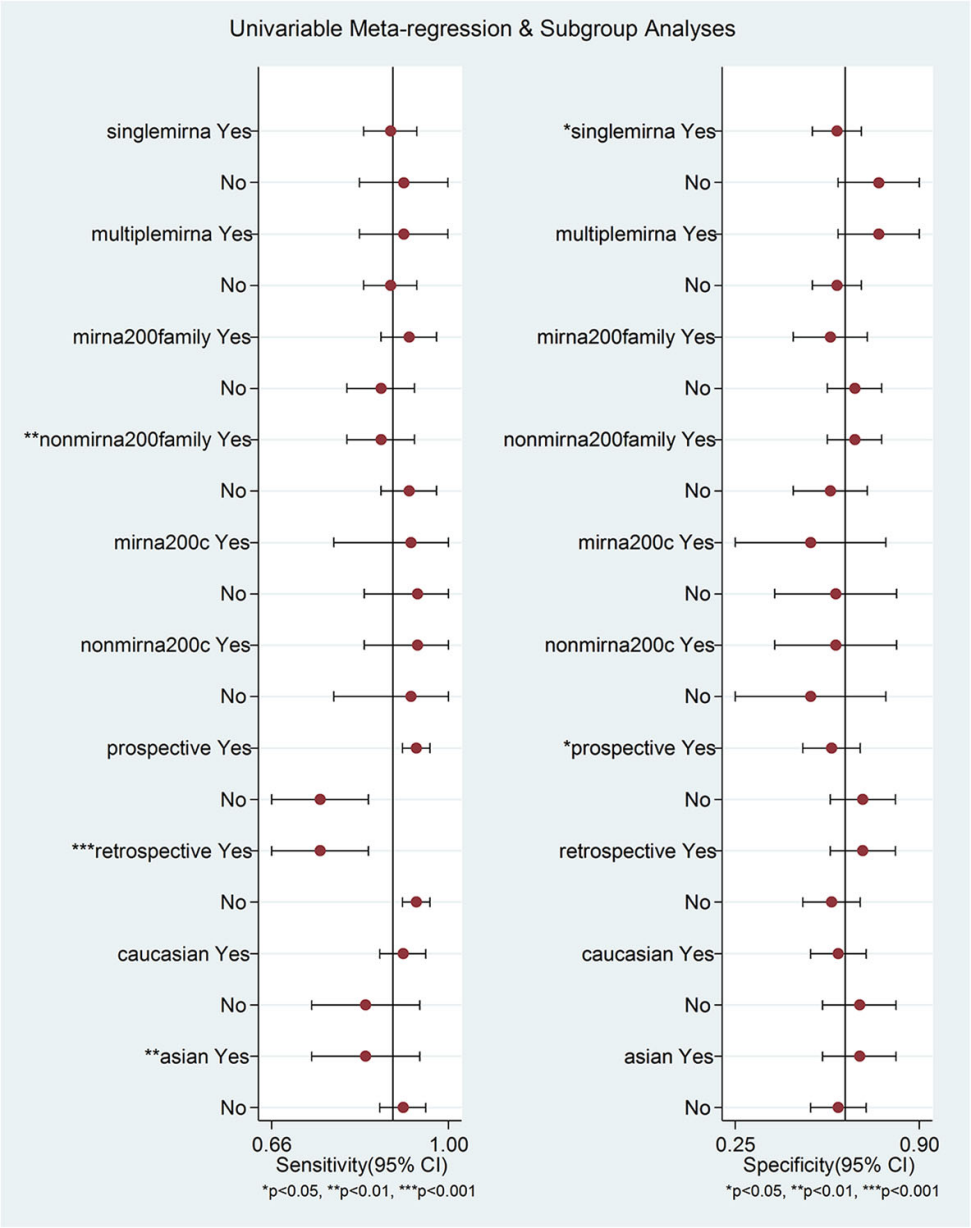
Meta-regression to explore heterogeneity between studies

Heterogeneity caused by differences in sensitivity and specificity is known as the threshold effect, which is an important source of heterogeneity in diagnostic tests. The correlation coefficient of logarithm sensitivity and specificity is a common approach to estimating a threshold effect. Our research data show that the correlation value was − 0.5001, revealing a threshold effect in this study. Moreover, we performed meta-regression to test the effect of ethnicity (Caucasian or Asian), study type (prospective or retrospective), and microRNA profiling (single or multiple miRNA, miRNA 200 or non-miRNA 200 family, and miRNA 200c or non-miRNA 200c) to explore heterogeneity sources (Fig. 5). Among these factors, single miRNA profiling, the miRNA 200 family, research type and Asian ethnicity differed significantly (*P* < 0.05). We then conducted subgroup analyses to explore the potential diagnostic values of the miRNAs detected.

**Fig. 6:**
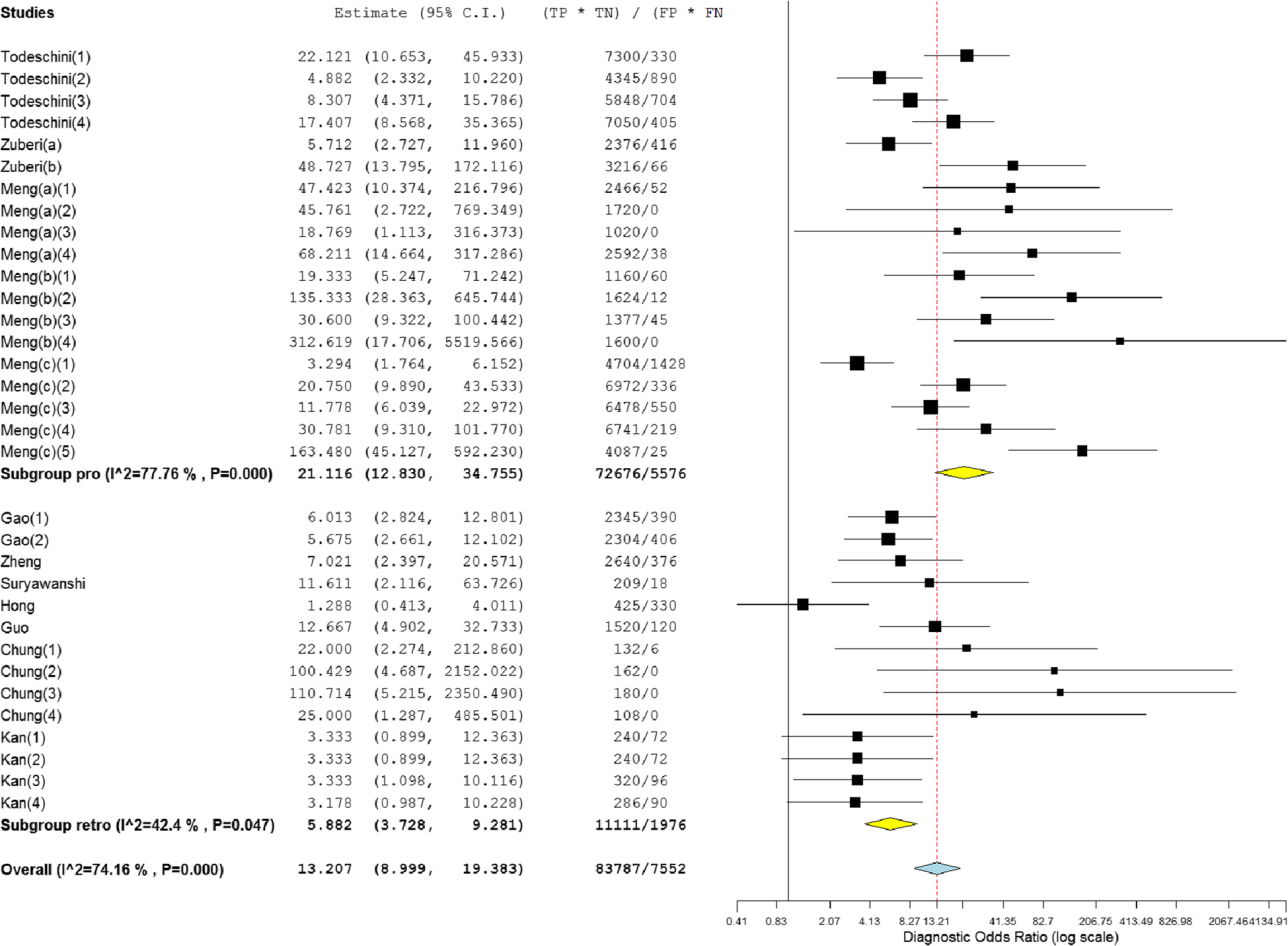
Subgroup analyses of diagnostic odds ratio (DOR) of miRNA assays by research design type

**Fig. 7:**
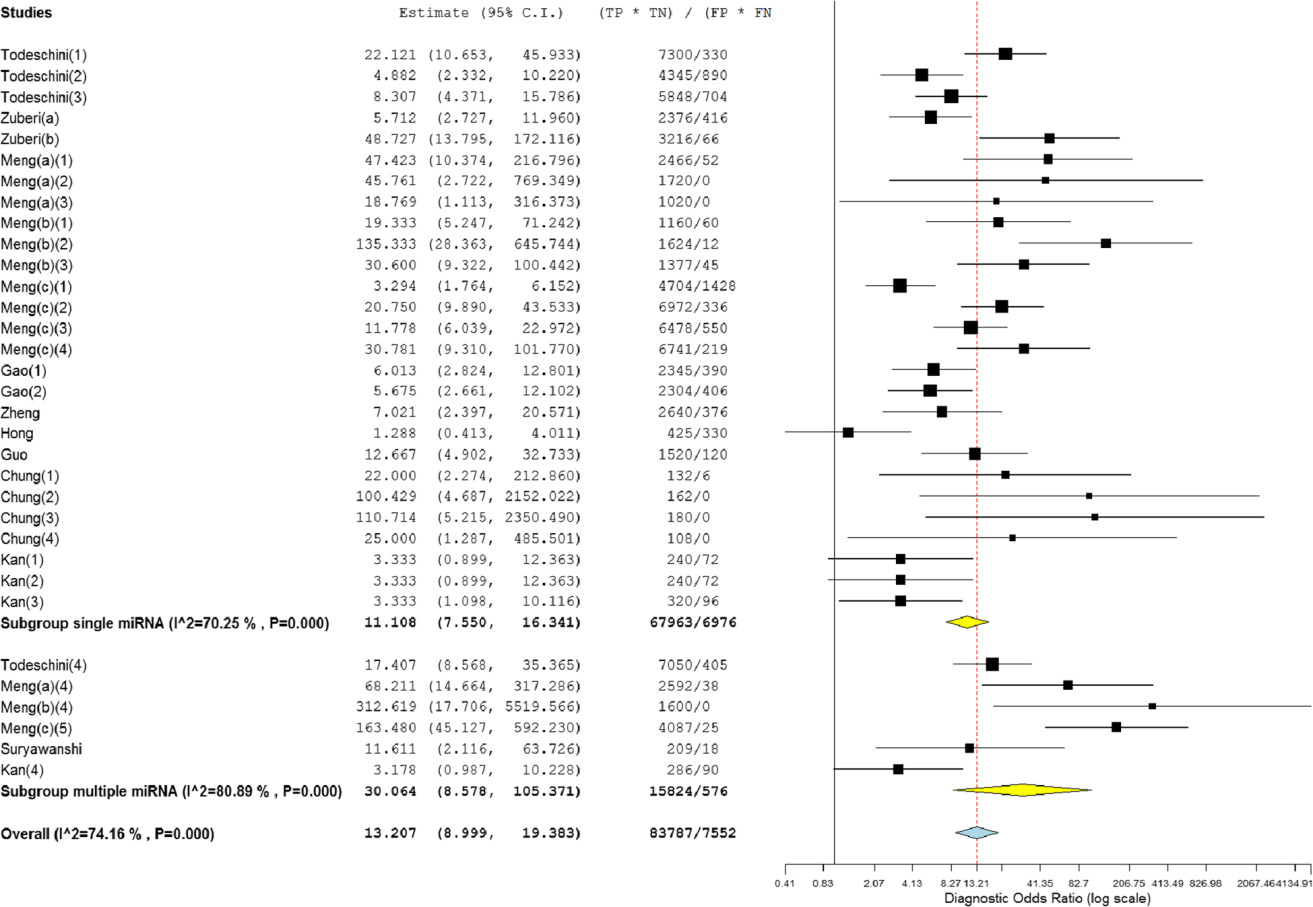
Subgroup analyses of diagnostic odds ratio (DOR) of miRNA assays between single miRNA and multiple miRNA panels

Subgroup analyses were performed on the research design type (prospective vs. retrospective). The results indicated that the prospective type (DOR, 21.12) more accurately detected miRNA than the retrospective type (DOR, 5.88) (Fig. 6). We also conducted subgroup analyses based on ethnicity (Caucasian vs. Asian), which indicated that miRNA detection accuracy in the Caucasian population (DOR, 15.28) was similar to that in the Asian population (DOR, 9.31) (Table 2). In addition, 6 datasets included multiple miRNA panels, and 27 datasets had single miRNAs. As shown in Fig. 7, the multiple-miRNA panel assays were more diagnostically accurate than the single miRNA assay, with the DOR increasing from 11.11 to 30.06. The summary assessments of miRNAs in diagnosing ovarian cancer are presented in Table 2.

**Table 2:** Summary of diagnostic criteria estimates and their 95% confidence intervals (95% CIs)

Subgroup	SEN (95%CI)	SPE (95%CI)	PLR (95%CI)	NLR (95%CI)	DOR (95%CI)
Type					
Pro	0.92 (0.89, 0.94)	0.59 (0.49, 0.68)	2.26 (1.89, 2.70)	0.10 (0.06, 0.15)	21.12 (12.83, 34.76)
Retro	0.73 (0.65, 0.79)	0.65 (0.55, 0.74)	1.98 (1.60, 2.45)	0.32 (0.23, 0.47)	5.88 (3.73, 9.28)
Ethnicity					
Asian	0.81 (0.74, 0.86)	0.64 (0.53, 0.74)	2.23 (1.71, 2.93)	0.23 (0.15, 0.35)	9.31 (5.03, 17.23)
Caucasian	0.89 (0.84, 0.92)	0.60 (0.51, 0.69)	2.16 (1.83, 2.55)	0.13 (0.08, 0.20)	15.28 (9.45, 24.68)
miRNA profiling					
Single miRNA	0.86 (0.81, 0.89)	0.59 (0.51, 0.66)	2.02 (1.79, 2.33)	0.17 (0.12, 0.26)	11.11 (7.55, 16.34)
Multiple miRNA	0.90 (0.75, 0.97)	0.74 (0.59, 0.85)	3.21 (2.06, 5.01)	0.09 (0.03, 0.28)	30.06 (8.58, 105.37)
miRNA 200 family	0.90 (0.80, 0.95)	0.58 (0.43, 0.72)	1.98 (1.61, 2.44)	0.11 (0.07, 0.17)	15.48 (7.15, 33.51)
miRNA 200c	0.85 (0.67, 0.94)	0.52 (0.22, 0.81)	1.88 (1.17, 3.03)	0.19 (0.06, 0.57)	8.84 (3.20, 24.40)
overall	0.89 (0.84, 0.93)	0.64 (0.56, 0.72)	2.18 (1.90, 2.51)	0.15 (0.11, 0.22)	13.21 (9.00, 19.38)

*SEN* sensitivity, *SPE* specificity, *PLR* positive likelihood ratio, *NLR* negative likelihood ratio, *DOR* diagnostic odds ratio, *miRNA* microRNA, *Pro* prospective, *Retro* retrospective.

### Sensitivity analysis and publication bias

**Fig. 8:**
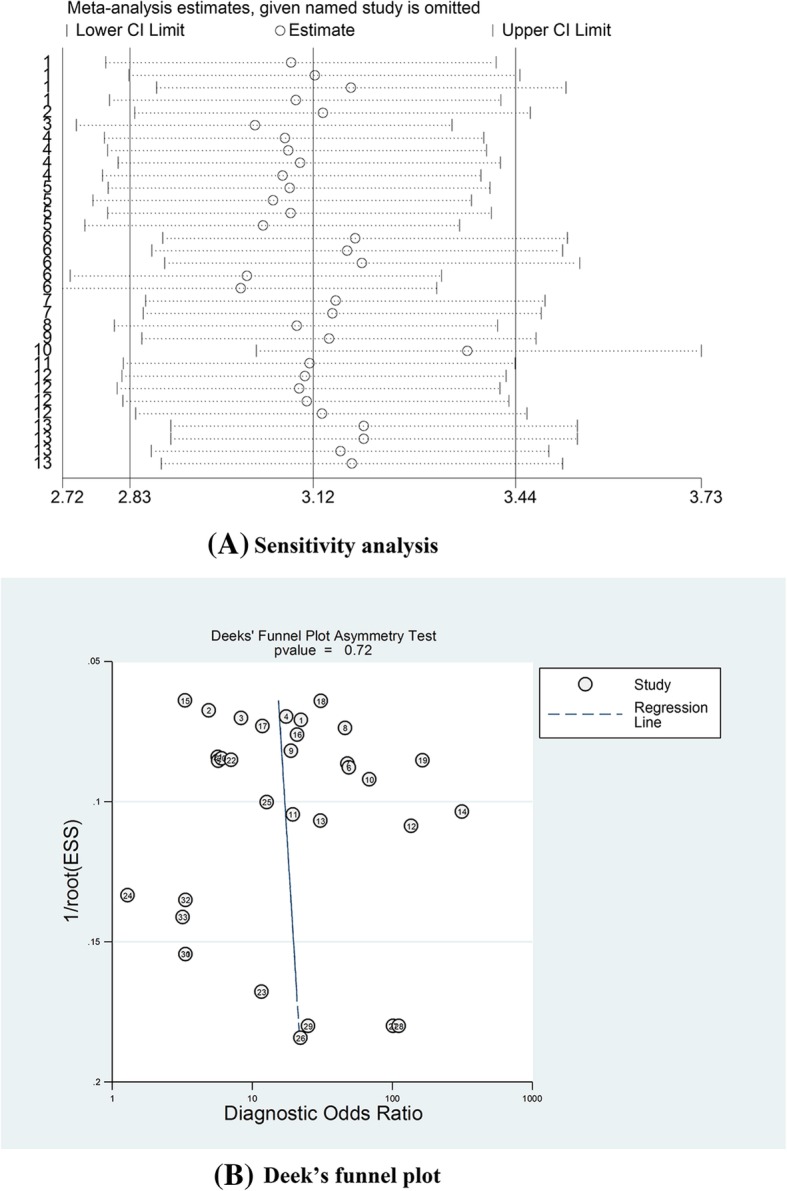
**a**. Sensitivity analysis to estimate each study’s value; **b**. Deeks’ funnel plot to assess potential publication bias

Sensitivity analysis was performed to ensure that our findings were not significantly affected by any individual study. The data shown in Fig. 8a suggest that each individual study had enough value to be used. In addition, no significant publication bias was detected by Deeks’ funnel plot asymmetry test (t = 0.36, *P* = 0.72) (Fig. 8b). The above tests confirmed the robustness of our meta-analysis results.

## Discussion

Ovarian cancer is the primary cause of cancer-associated death among gynaecological malignancies, representing 5% of all cancers in women [1]. While pelvic examinations, transvaginal ultrasonography, and serum CA125 tests are performed during routine diagnostic procedures, they are insufficiently sensitive and specific for making early diagnoses [33]. Serum CA125, which is considered a promising non-invasive tumour biomarker, revealed a pooled sensitivity of 0.80 for diagnosing ovarian cancer as well as a 0.75 specificity with moderate diagnostic value [34]. Recently, microRNA has drawn researchers’ attention due to its significant roles in tumour genesis and progression. Many studies on the diagnostic value of microRNA have emerged; however, the results are debatable because of the different study designs used [35]. Thus, we conducted this meta-analysis to identify the feasibility and overall diagnostic value of the miRNA tests.

In this meta-analysis, as a diagnostic biomarker, circulating miRNAs had a moderate diagnostic accuracy and achieved a combined DOR of 13.21, with a 0.89 pooled sensitivity and 0.64 pooled specificity, in discriminating ovarian cancer. As an advantage over a single indicator, the pooled DOR was 13.21, with higher values indicating better discriminatory test performance. We also found that the likelihood ratios (PLR and NLR) are more clinically meaningful for measuring diagnostic accuracy. In our meta-analysis, we found an NLR value of only 0.15, which refers to the probability of a person with ovarian cancer testing negative, divided by the probability of a person without ovarian cancer testing negative. These results revealed that circulating miRNAs can clearly distinguish ovarian cancer patients from healthy patients.

As the study’s heterogeneity was large, we adopted the bivariate random effects model to reduce heterogeneity-related bias. Through the correlation value, we found heterogeneity to be a result of the threshold effect. The results from our meta-regression suggested that ethnicity, design type, and microRNA profiling might be sources of heterogeneity; therefore, subgroup analyses were used to investigate and explain the heterogeneity. The results suggested that circulating miRNAs could be used as ovarian cancer diagnostic biomarkers in both Caucasian and Asian populations with moderate diagnostic accuracy. Further, the Caucasian and Asian populations did not significantly differ. The diagnostic value of the research types varied considerably. Prospective studies were more reliable than retrospective studies and had more evidence with a relatively high diagnostic accuracy and a combined DOR of 21.12, suggesting that the miRNAs’ actual diagnostic value might be higher than that of the overall value. In addition, we conducted a subgroup analysis based on the number of miRNAs being profiled. The results suggested that multiple miRNA panels were more accurate with a combined DOR of 30.06 in detecting ovarian cancer. These results revealed that multiple miRNA panels more clearly distinguished ovarian cancer patients from healthy individuals, and more comprehensive prospective research may gain insight into the clinical value of miRNAs in the future.

Compared with previous similar studies, comprehensive analysis of the diagnostic value of circulating miRNAs in ovarian cancer patients found that the pooled sensitivity was 0.75, specificity was 0.75, and the AUC was 0.82 [36]. Diagnostic meta-analysis showed a reliable diagnostic capacity for miR-200c-3p (with an AUC of 0.77) for epithelial ovarian cancer [37]. Our present meta-analysis also showed similar results and verified that circulating miRNAs had a relatively high diagnostic accuracy. Furthermore, the advantages of our meta-analysis are as follows. First, this study was more comprehensive and detailed, with 21 more datasets than the previous meta-analysis. In addition, more prospective studies were included, which enhanced the study’ reliability. Second, due to the different diagnosis cut-off values and different potential factors leading to heterogeneity in the same diagnostic test, heterogeneity was significant among the different studies in each study. Compared with previous studies, a bivariate random effects model was used to analyse the data in this study, which preserved the two-dimensional characteristics of the raw data, reduced the bias from the threshold effect, and increased the results’ reliability. Third, to explore the sources of heterogeneity, subgroup and meta-regression analyses were performed to investigate the diagnostic performance of miRNAs in ovarian cancer. Subgroup analyses revealed that multiple miRNA panels were recommended to detect ovarian cancer. Finally, in our meta-analysis, all articles independently conducted quality assessments and collated cases and controls from every study, which significantly improved statistical efficacy.

Although this meta-analysis yielded promising results, several limitations must be addressed. First, the biological characteristics and mechanisms of the different miRNAs in ovarian cancer may differ, and only three miRNA markers (miRNA 200a, 200b, 200c) were repeated. In addition, methods for accurately and absolutely quantifying miRNAs are not uniformly normal, which limited applicability of the pooled analysis. Second, our data suggest that multiple miRNA panels can achieve better accuracy; however, the numbers required and the most efficient method for combining the miRNA panel remains unreported. Therefore, a rational and efficient miRNA panel should be investigated to improve future miRNA assay outcomes.

## Conclusion

Despite these limitations, our meta-analysis demonstrated that circulating miRNAs may be novel and non-invasive biomarkers for detecting ovarian cancer, especially multiple miRNA panels, which have potential diagnostic value as screening tools for clinical practice. Further large-scale prospective studies are warranted to improve the accuracy and explore the most effective miRNA combinations.

## Additional file

Additional file 1: Additional Supporting Information may be found in the online version of this article. (DOCX 20 kb)
