# Supplementary material for: Circulating microRNAs as novel potential diagnostic biomarkers for ovarian cancer: a systematic review and updated meta-analysis
Source: J Ovarian Res. 2019 Mar 21;12:24. doi: 10.1186/s13048-019-0482-8 (PMC6427862; doi:10.1186/s13048-019-0482-8)
Supplement: Supplementary file 1 — Additional Supporting Information may be found in the online version of this article. (DOCX 20 kb) [file 13048_2019_482_MOESM1_ESM.docx]

**Search details**

**EMBASE**

**Searches Results**

1 Ovarian cancer.mp. or exp ovary cancer/ 110577

2 exp microRNA/ or microRNA.mp. 102511

3 diagnosis.mp. or exp diagnosis/ 7342313

4 sensitivity.mp. 1192337

5 specificity.mp. 755389

6 ROC curve.mp. or exp receiver operating characteristic/ 91635

7 3 or 4 or 5 or 6 8381545

8 1 and 2 and 7 837

**Pubmed**

Query

1 Search (((((((((Ovarian Neoplasms) OR Ovarian Neoplasm) OR Ovary Neoplasms) OR Ovary Neoplasm) OR Ovary Cancer) OR Ovary Cancers) OR Ovarian Cancer) OR Ovarian Cancers) OR Cancer of Ovary) OR Cancer of the Ovary

[110620](https://www.ncbi.nlm.nih.gov/pubmed/?cmd=HistorySearch&querykey=3)

2 Search ((((((((((((MicroRNAs) OR MicroRNA) OR miRNAs) OR Micro RNA) OR miRNA) OR Primary MicroRNA) OR Primary miRNA) OR pri-miRNA) OR pri miRNA) OR stRNA) OR Small Temporal RNA) OR pre-miRNA) OR pre miRNA

[71783](https://www.ncbi.nlm.nih.gov/pubmed/?cmd=HistorySearch&querykey=8)

3 Search (((ROC Curve) OR diagnos*) OR specificity) OR sensitivity

[5468807](https://www.ncbi.nlm.nih.gov/pubmed/?cmd=HistorySearch&querykey=9)

4 Search ((((((ROC Curve) OR diagnos*) OR specificity) OR sensitivity)) AND (((((((((((((MicroRNAs) OR MicroRNA) OR miRNAs) OR Micro RNA) OR miRNA) OR Primary MicroRNA) OR Primary miRNA) OR pri-miRNA) OR pri miRNA) OR stRNA) OR Small Temporal RNA) OR pre-miRNA) OR pre miRNA)) AND ((((((((((Ovarian Neoplasms) OR Ovarian Neoplasm) OR Ovary Neoplasms) OR Ovary Neoplasm) OR Ovary Cancer) OR Ovary Cancers) OR Ovarian Cancer) OR Ovarian Cancers) OR Cancer of Ovary) OR Cancer of the Ovary

[408](https://www.ncbi.nlm.nih.gov/pubmed/?cmd=HistorySearch&querykey=10)

**Cochrane**

#1 MeSH descriptor: [Ovarian Neoplasms] explode all trees 1527

#2 Ovarian Neoplasm or Ovary Neoplasm or Ovary Cancer or Ovarian Cancer (Word variations have been searched) 5096

#3 MeSH descriptor: [MicroRNAs] explode all trees 176

#4 MicroRNA or miRNA or Micro RNA or stRNA (Word variations have been searched) 620

#5 ROC Curve or diagnosis or diagnostic or specificity or sensitivity (Word variations have been searched) 180063

#6 #1 or #2 5118

#7 #3 or #4 677

#8 #5 and #6 and #7 12

**Chinese database**

CNKI 29

Wanfang 38

VIP 36
